# The Significance of Echo Time in fMRI BOLD Contrast: A Clinical Study during Motor and Visual Activation Tasks at 1.5 T

**DOI:** 10.3390/tomography7030030

**Published:** 2021-08-05

**Authors:** Themistoklis Boursianis, Georgios Kalaitzakis, Katerina Nikiforaki, Emmanouela Kosteletou, Despina Antypa, George A. Gourzoulidis, Apostolos Karantanas, Efrosini Papadaki, Panagiotis Simos, Thomas G. Maris, Kostas Marias

**Affiliations:** 1 Department of Medical Physics, School of Medicine, University of Crete, 71003 Heraklion, Greece; g.kalaitzakis@med.uoc.gr (G.K.); marist@uoc.gr (T.G.M.); 2 Computational Biomedicine Laboratory (CBML), Institute of Computer Science, Foundation for Research and Technology—Hellas (FORTH), 70013 Heraklion, Greece; kat@ics.forth.gr (K.N.); karantanas@uoc.gr (A.K.); fpapada@uoc.gr (E.P.); simosp@uoc.gr (P.S.); kmarias@ics.forth.gr (K.M.); 3 Institute of Applied Mathematics, FORTH, 70013 Heraklion, Greece; emma_kost@hotmail.com; 4 Department of Psychiatry, School of Medicine, University of Crete, 71003 Heraklion, Greece; despina.antypa@gmail.com; 5 Research & Measurements Center of OHS Hazardous Agents, OHS Directorate, Hellenic Ministry of Labor, 10110 Athens, Greece; ggourz@ypakp.gr; 6 Lighting Lab, National Technical University of Athens, 15780 Athens, Greece; 7 Department of Radiology, School of Medicine, University of Crete, 71003 Heraklion, Greece; 8 Department of Electrical and Computer Engineering, Hellenic Mediterranean University, 71410 Heraklion, Greece

**Keywords:** MR imaging, T2* measurement, echo time, BOLD, fMRI/visual activation, fMRI/motor activation

## Abstract

Blood Oxygen Level Dependent (BOLD) is a commonly-used MR imaging technique in studying brain function. The BOLD signal can be strongly affected by specific sequence parameters, especially in small field strengths. Previous small-scale studies have investigated the effect of TE on BOLD contrast. This study evaluates the dependence of fMRI results on echo time (TE) during concurrent activation of the visual and motor cortex at 1.5 T in a larger sample of 21 healthy volunteers. The experiment was repeated using two different TE values (50 and 70 ms) in counterbalanced order. Furthermore, T2* measurements of the gray matter were performed. Results indicated that both peak beta value and number of voxels were significantly higher using TE = 70 than TE = 50 ms in primary motor, primary somatosensory and supplementary motor cortices (*p* < 0.007). In addition, the amplitude of activation in visual cortices and the dorsal premotor area was also higher using TE = 70 ms (*p* < 0.001). Gray matter T2* of the corresponding areas did not vary significantly. In conclusion, the optimal TE value (among the two studied) for visual and motor activity is 70 ms affecting both the amplitude and extent of regional hemodynamic activation.

## 1. Introduction

The significance of Blood Oxygen Level Dependent (BOLD) response for non-invasively defining activated regions during performance of sensorimotor or cognitive tasks, is indisputable for optimal surgical planning and understanding brain function [1,2]. EPI has been the most commonly-used method in clinical practice since the 1990s when it was first introduced. The establishment of an optimal protocol is of utmost importance in order to approximate the degree and extent of underlying neurophysiological activity.

Several studies have explored the impact of field strength and parameters of the BOLD sequences on BOLD sensitivity [3,4,5,6,7,8,9]. Especially at lower field studies (1.5 T) determining the conditions for obtaining the highest possible fMRI signal is of great importance in order to reach reliable conclusions. Noise in fMRI is physiological, arising from metabolic activity and brain pulsations, as well as thermal and electrical, associated with scanner electronics [10]. Metabolically-linked physiological noise, in contrast to other sources of noise, renders echo time particularly important in fMRI studies. It has been shown that the optimal setting for achieving the highest possible signal, is an Echo Time (TE) equal to tissue T2* relaxation constant [11,12]. T2* may vary among different anatomical locations due to the different iron and myelin content as well as vascularity of the region producing variable susceptibility induced spin dephasing [13].

In EPI acquisitions, a dephasing gradient is applied initially forming a gradient echo followed by consecutive blips of the opposite polarity in the phase-encode direction. A series of gradient echoes is formed by reversing the polarity of the blipped gradient. TE in EPI acquisition is the effective TE defined as the time between the RF excitation pulse to the center of the echo train. The signal detected in each of these echoes is subject to the T2* decay envelope and thus longer echo times entail reduced available detectable signal. However, overall available signal does not necessarily mean more fMRI related signal as only the excess signal in the activation areas compared to the rest state is relevant for these studies and not the total signal received by the coil. The portion of fMRI signal does not exceed 1–3% of the overall signal at 1.5 T.

Previous work, often with small sample sizes, suggested that the magnitude of the effect of TE may be variable across brain regions [4,6,8,9,13,14,15]. For instance, van der Zwaag et al. [6] focused on the primary motor cortex of 6 participants showing that significantly more activated voxels appeared when TE was set at 70 ms as compared to TE = 50 ms (at 1.5 T). Donahue et al. [8] focused on the primary visual cortex of 7 volunteers and found the same number of activated voxels either using TE = 53 ms or 73 ms (again, at 1.5 T). Although the measured T2* value is expected to vary considerably between MRI systems, the association between actual, measured T2* values from the healthy cerebral cortex on the impact of TE was not explicitly assessed in previous studies.

The aim of the present study was two-fold. Firstly, to establish the impact of TE on the sensitivity of BOLD activation paradigms and, secondly, to comprehend the complex relationship between TE and brain tissue T2* value. To address these goals, we performed a simultaneous visual and motor fMRI experiment conjoined with a T2* relaxometry study of the brain. In a repeated measures design, each participant performed the blocked fMRI task twice (in counterbalanced order across participants): once with a TE close to calculated grey matter T2* (70 ms) and one with the lower TE conventionally implemented in fMRI studies (50 ms). The task involved alternating fixation (baseline) and visuomotor blocks. The latter involved visual stimulation with a phase-alternating black and white checkerboard during which participants were asked to perform self-paced finger movements. In this manner the effect of TE on activation parameters in both primary and secondary visual and sensorimotor areas could be studied simultaneously, controlling for signal fluctuations due to MRI system instabilities. Finally, T2* relaxometry measurements were obtained from the same participants to explore potential, subtle effects of tissue T2* to the detected activation. To the authors’ best knowledge, an fMRI acquisition with a double activation task for the study of the effect of TE on BOLD signal in two different brain areas has not been previously studied.

## 2. Materials and Methods

### 2.1. Participants

The fMRI data were obtained from 21 healthy adults (mean age = 27.8 ± 5.6; 11 males and 10 females) without any history of neurological or psychiatric disorders. They all had normal or corrected to normal vision and provided written consent in accordance to the declaration of Helsinki. The study was approved by the Research Ethics Committee of the University of Crete, Protocol number: 138/09.07.2019.

### 2.2. Stimuli and Tasks

Participants completed two identical observation and action tasks simultaneously, acquired with two different values of TE (50 and 70 ms) and presented in counterbalanced order. Each task comprised three baseline blocks of 20.65 s each, alternating with three active blocks of 20.65 s each. Active blocks consisted of the presentation of an alternating checkerboard (8 Hz) during which the participants were instructed to perform a sequential finger tapping (i.e., tapping their right-hand thumb with each other finger of the right hand, one after the other). The stimulus set up was identical across blocks and tasks. Baseline blocks consisted of a blank screen with a white cross located at the center of the screen for the entire duration of the recording. During baseline blocks participants were asked to focus on the cross and simultaneously stop the finger tapping and keep their hand as still as possible.

### 2.3. MR Image Acquisition

A whole-body clinical MRI system 1.5 T (MAGNETOM Sonata/Vision Hybrid, Siemens Healthcare, Erlangen, Germany), equipped with a gradient system (Gradient maximum strength: 40 mT/m, Gradient rise time: 200 μs, Gradient Slew rate: 200 mT/m/ms) was utilized. A standard quadrature RF bird cage body coil was used for signal excitation and a 2-channel, 2-loops, circular polarization head coil (from the same vendor) was used for signal detection. Parallel imaging was not possible with the coil used.

Whole brain scans consisted of 36 slices of 3 mm thickness and no interslice gap. The slice group orientation was an oblique axial plane aligned parallel to the anterior-posterior commissure line.

### 2.4. Anatomical/Diagnostic

High resolution anatomical images were acquired using a 3D magnetization-prepared rapid acquisition gradient echo sequence (3D-MPRAGE). The acquisition time was 4 min and 23 s. Additionally, a 2D-TSE-FLAIR sequence was performed and the absence of any congenital anatomic variations or unexpected pathology was confirmed. The acquisition time was 2 min and 8 s.

### 2.5. Relaxometry

For the quantitative measurement of T2*, a Multi-Echo-Gradient-Echo (MEGRE) sequence consisting of 12 echoes was performed. The acquisition time was 2 min 6 s.

### 2.6. Functional

For the BOLD-fMRI, a T2*w fat-saturated 2D-GRE-EPI sequence was used. Each BOLD time series was 4 min and 13 s in duration and consisted of 60 dynamic volumes. All the sequences parameters are presented in Table 1.

### 2.7. Preprocessing

Image preprocessing was performed with SPM12. At first, EPI scans were spatially realigned to the mean image of the time-series using second-degree Bspline interpolation algorithms and motion-corrected through rigid body transformations (three translations and three rotations about each axis). Next, images were spatially normalized to a common brain space (MNI template), smoothed using an isotropic Gaussian filter (FWHM = 8 mm), and high pass filtered with a time constant of 128 s.

Afterwards, the preprocessed signal was analyzed directly using a fixed effect General Linear Model (GLM) [16] in SPM12, separately for each participant and task (two different TEs). The model included two condition regressors of interest (active, baseline), six motion regressors of no-interest. Resulting T values were thresholded at *p* < 0.05 FWE-corrected with a minimum cluster size of 20 voxels.

### 2.8. Strength of Activation

First-level statistical T maps thresholded at *p* < 0.001, uncorrected, and derived from each of the two conditions (TE50, TE70) were overlaid to identify common regions of activation (task > baseline). Such clusters were found in 6 regions in all participants: primary visual cortex along the banks of the calcarine sulcus (BA17), ventral extrastriate cortex (BA18), primary motor cortex in the vicinity of the hand area (BA4), primary somatosensory cortex (hand area; BA2/3), dorsal premotor cortex (BA6), and supplementary motor cortex (BA6). Next, the center of a 5-mm radius spherical ROI was placed at the geometric center of each of the activated voxel clusters. The peak beta value across all voxels within each spheroid for a given condition and participant served as the dependent variable indicating strength of activation in relation to the fixation baseline.

### 2.9. Extent of Activation within Each Activated Anatomical Region

The number of suprathreshold voxels (at *p* < 0.001, uncorrected in first-level analyses) within each of the 6 anatomical areas, which hosted activation clusters in both conditions, served as the second dependent variable of the study. Voxel counts were performed by computing the intersection of each voxel cluster with the following sets of regions from the automated anatomical labelling (aal) atlas. The left precentral gyrus mask was used to identify active voxels in the primary motor cortex, the left postcentral gyrus mask for the primary somatosensory cortex, the left superior frontal gyrus mask for the dorsal premotor cortex (with one exception where the corresponding right hemisphere mask was used instead). Given the location of SMA where even slight coregistration errors can cause activity voxels to be placed in the opposite hemisphere, we used both the right and left supplementary motor area masks. With respect to primary visual cortices we used four aal area masks to accommodate bilateral activation with the borders of BA 17 (calcarine and cuneus). A similar decision was made regarding ventral extrastriate cortex where the lingual gyrus mask was applied bilaterally: although activation was detected only in the right hemisphere or had larger amplitude in that hemisphere (in 15/21 participants) there were substantial clusters in the homologous left hemisphere areas in 13/21 participants.

### 2.10. Quantitative T2* Analysis

The T2* parametric maps were constructed on a pixel by pixel basis on a separate workstation utilizing an in-house software (QMRI utilities—X). The T2* values were sampled at multiple sites within two broad regions covering the areas where significant activation was detected in the present study, namely the posterior occipital lobe and the posterior portion of the frontal lobe. The ROIs were manually drawn by a neuroradiologist (E.P.), with 20 years of experience, on the T1w sequence. Using a freehand tool, only the gray matter of the examined brain region was selected. In order to accurately transfer the drawn ROI, T2* maps were co registered on the anatomical T1w images using Nordic Ice software (Nordic Neurolab, Bergen, Norway).

### 2.11. Statistical Analyses

This main research question was addressed via repeated measures ANOVAs comparing the two sequences (TE = 50 vs. TE = 70 ms) on each of the two dependent variables (number of voxels and peak beta value in each ROI). The significance level was set to *p* < 0.008 using Bonferroni correction for 6 comparisons.

## 3. Results

**Figure 1:**
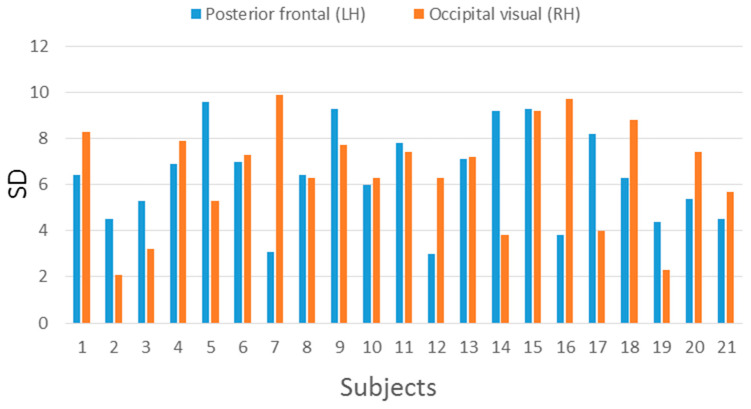
Variability of T2* measurements (indexed by the SD across multiple sampling sites) across participants and regions of interest.

**Figure 2:**
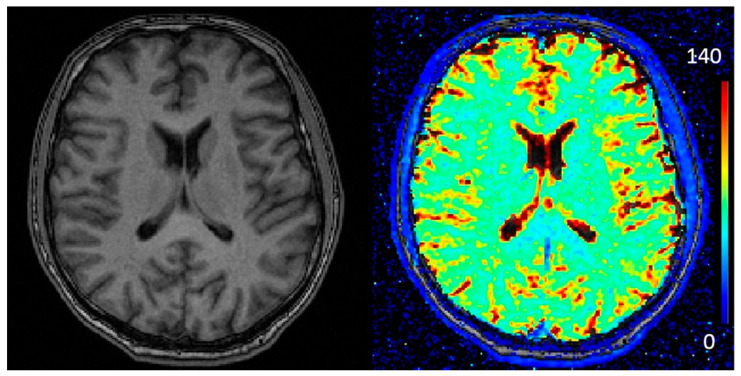
T1w image (**left**) and T2* (ms) parametric map (**right**) from a selected participant.

The ROI T2* measurements (mean, SD) for each subject are presented in Table 2. The lowest T2* value of 53.10 ms (SD 2.1) was observed in visual cortex of subject 2. Accordingly, the highest T2* value of 79.75 ms (SD 7.4) was observed in visual cortex of subject 20. All SDs are presented in color bars (Figure 1). However, the degree of variability within a given ROI (across sampling sites) was very comparable to the degree of variability between ROIs for a given person, implying significant inhomogeneity in the distribution of T2* values over the cortical surface. Global average T2* values ranged between 64 and 79 ms (Mean = 71.7 ms, SD = 3.3). T2* colormap and T1w image from a selected patient are shown in Figure 2.

**Figure 3:**
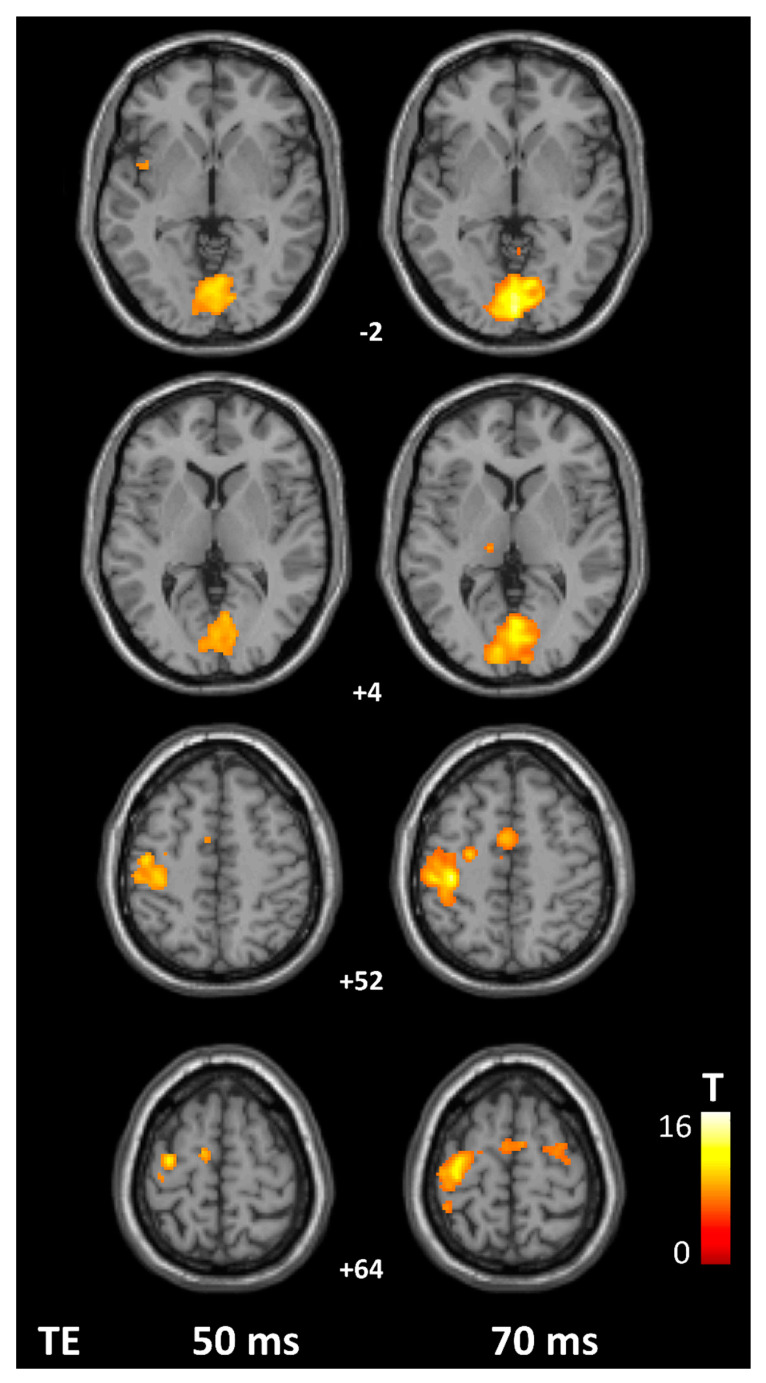
Group-level activation maps by TE condition, thresholded at *p* < 0.05 FWE. V2 activity appears primarily in the uppermost panel, V1 in the upper-middle panel (bilaterally), left MI, SI and bilateral SMA in the lower-middle panel, whereas left PMd activation is apparent in the lowest panel. Numbers represent z MNI coordinates (in mm).

Group-level activation maps by TE condition are displayed in Figure 3 (thresholded at *p* < 0.05, FWE-corrected, with minimum cluster size of 20 voxels). Use of a longer TE significantly improved beta values in all 6 ROIs (*p* < 0.001), and resulted in higher number of active voxels in the left MI (*p* = 0.002), SI (*p* = 0.007), and bilateral SMA (*p* = 0.003; Table 3).

**Figure 4:**
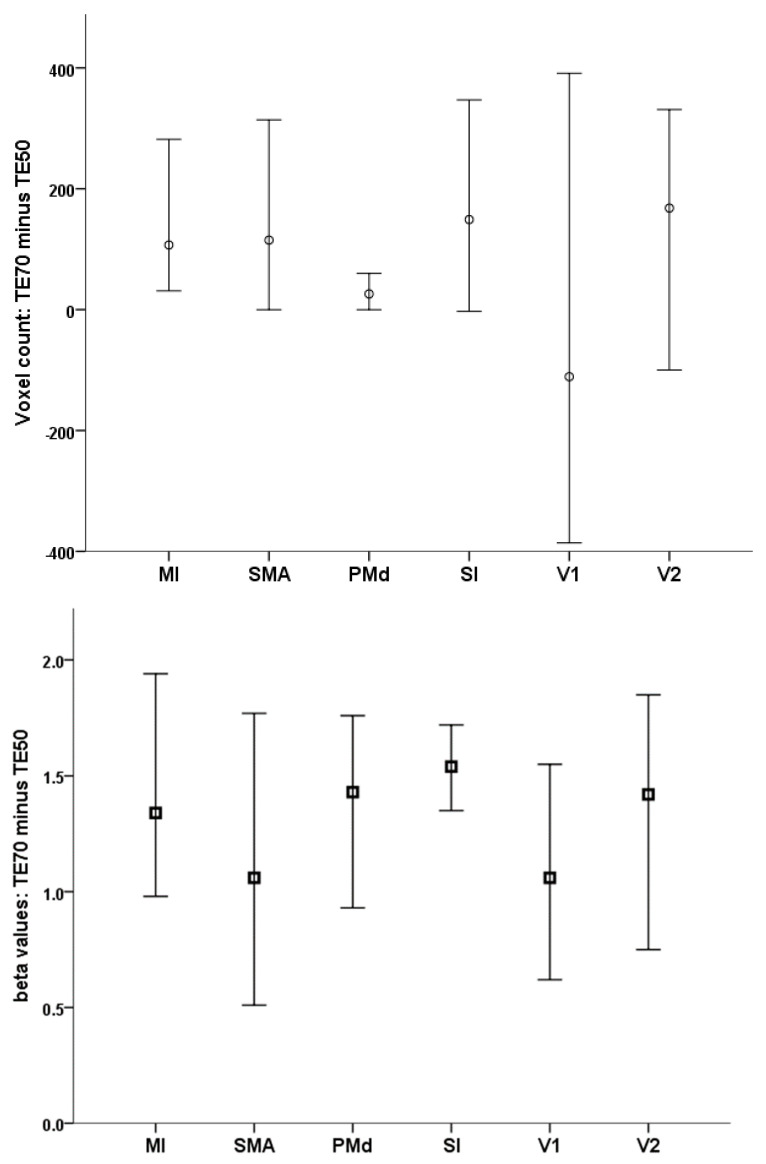
Difference in voxel number (**upper panel**) and in peak beta values (**lower panel**) in the 6 brain regions where significant task-related activity was found in the present study. Vertical bars represent 95% confidence intervals.

These differences were associated with large effect sizes as indicated by η^2^ values (0.31 > η^2^ > 0.78). In terms of peak beta values, effect sizes were very similar across ROIs (η^2^ = 0.57–0.78). Parametric maps of activation obtained from second level analyses (group maps, thresholded at *p* < 0.05 FWE) are shown in Figure 3. As illustrated in Figure 4, these effects were apparent in individual activation maps: Peak beta values increased in 21/21 scans in MI and SI, and in 20/21 participants in SMA and PMd, V2, and in 19/21 participants in V1. Sphere center coordinates for each region and participant are presented in Supplementary Table S1.

Regarding the second research aim of the present study, the results were not conclusive, given that we did not find significant correlations between individual, ROI-specific T2* values and degree of change in either amplitude of extent of activation in the 50 vs. 70 ms TE condition.

## 4. Discussion

In this study the effect of the chosen TE on the recorded BOLD signal was evaluated in a relatively large sample of 21 healthy volunteers at 1.5 T. According to our results, the optimal TE value for visual and motor activity was 70 ms (compared to 50 ms, commonly used in fMRI studies), affecting both the amplitude and extent of regional hemodynamic activation. Peak beta value and number of voxels were significantly higher using a TE of 70 ms than 50 ms in primary motor, primary somatosensory and supplementary motor cortices. Results indicated that both the amplitude of activation in visual cortices and the dorsal premotor area was also higher using a TE of 70 ms.

EPI BOLD sequences have been deployed for functional MRI due to their sensitivity in estimating regional neuronal activation and high speed of acquisition compared to other sequences, i.e., SE BOLD. In such experiments, especially for lower field MRI, it is crucial to achieve the optimal balance between a long TE, which would in principle increase the recorded BOLD signal, and at the same time satisfactory SNR, in order to correctly assign signal changes to blood flow alterations. Moreover, considering that the extent of the activated area is largely dependent on user-defined thresholds, it is important to rely on a robust acquisition scheme that will provide well-adjusted representations of underlying neurophysiological activity. Another potential confound in the fMRI signal could be the inflow effect when Gradient Echo techniques (with T1 weighting and relatively short TR) are used for BOLD acquisition. However, the implemented EPI sequence acquired the whole brain volume (36 slices) in a single shot, with a TR of 4130 ms. Inflow effects are, therefore, negligible in such setting even when a large flip angle is used [17].

T2* measurements performed in the occipital and frontal gray matter were in agreement with previously reported values [18]. There was considerable variability in T2* measurements within each region which was rather uniform across participants and brain regions (Figure 1). The observed differences in brain tissue T2* can be attributed to different myelin or iron concentration [19]. It should be noted that average gray matter T2* did not vary significantly between the two lobar sub-regions where the vast majority of activity clusters were detected in the present study (posterior frontal and occipital).

Our results indicated that improvement in signal detectability (indexed by beta values) was strongly and positively related to the TE setting in MΙ (and to a lesser extent in V1). These results are in agreement with previous, smaller scale studies [6,8], which examined either only MΙ or V1. Importantly, detectability of activation in terms of strength of activation was also affected in non-primary motor cortex (SMA). Even more, the extent of activation was increased at TE = 70 ms in all areas where reliable activation was observed across TE conditions. This applies to the primary and extrastriate visual cortex, as well as primary sensorimotor and premotor cortices. Although the present study did not assess this scanning parameter, the effect of TE was demonstrated in sequences employing a relatively small number of volumes (60). The significance of the above findings, is further highlighted by the high degree of consistency of the effect of TE on a case-by-case basis which was, again, noted in several key regions of the visual and sensorimotor circuits.

In interpreting these results, one should consider that apart from neuronal activity, the choice of TE also affects vascular contributions to the BOLD signal. Shorter TE settings favor contributions from intravascular components, whereas the venous signal is weaker at longer echo time acquisitions. Other factors, such as vessel size and orientation also affect the amount of detected signal. It is evident that the detected signal is the result of a complex interplay between neuronal activity and vasculature [15] and it is difficult to decompose the effect of each factor independently, on the commonly used EPI BOLD sequences.

Overall, these results suggest that in motor tasks one should use a longer TE to improve sensitivity in detecting the amplitude of activity in both primary sensorimotor as well as association motor cortices. In the context of visual tasks, however, measuring T2* values in occipital cortices and adjusting the sequence TE to be as close as the measured T2* value, appears to be less beneficial. However, increasing TE requires a greater TR, which in turn affects total scan time. In our study, the implemented sequence with TE = 50 ms, permits use of a shorter TR (3500 ms instead of 4130 ms) and thus a shorter scan time by up to 15%. For a fair comparison though, the TR = 4130 ms was used in both sequences.

Although, the significance of TE for fMRI BOLD contrast was evaluated, there are limitations in this study that should be mentioned. An implementation of a multi echo BOLD sequence was not possible in the MRI system used in this study. This approach would be beneficial in order to establish the optimum TE by evaluating a wider range of TE values (i.e., lower as well as higher than the measured brain T2* values). Moreover, average T2* measurements for all subjects were obtained only from the gray matter of two extensive cortical regions (posterior frontal, posterior occipital lobe). Future work could obtain detailed regional whole brain T2* measurements.

## 5. Conclusions

In conclusion, a TE value of 70 ms was proved to be better than the commonly used TE of 50 ms for the visual and motor activity in fMRI studies, affecting both the amplitude and extent of regional hemodynamic activation at 1.5 T. Taking into consideration that T2* measurements depend upon the local system or patient-induced field inhomogeneities, this study also suggests that it would be beneficial to obtain brain T2* values under the same imaging conditions (hardware/patient position etc.) and set the TE value at or near the reference T2* to improve BOLD contrast in subsequent fMRI experiments.

## Figures and Tables

**Table 1:** Sequence parameters.

Parameters	2D TSE-FLAIR	2D T2*w MEGRE	3D T1w MPRAGE	2D GRE EPI (BOLD)
Field Of View (mm^2^)	192 × 192	192 × 192	192 × 192	192 × 192
Acquisition Matrix	192 × 192	128 × 128	192 × 192	64 × 64
Slice orientation	Oblique axial AC-PC line	Oblique axial AC-PC line	Oblique axial AC-PC line	Oblique axial AC-PC line
Slice thickness (mm)	3	3	3	3
Flip angle (°)	150	25	15	90
Pixel Bandwidth (Hz/Pixel)	200	450	230	3255
Partial Fourier	Phase: off	Phase: 6/8	Phase: 7/8, slice: off	Phase: off
Repetition Time (ms)	10,000	1300	778	4130
Echo Time (ms)	92	2.4 × (*n* + 1) *n* = 0 to 11	272	50/70
Inversion Time (ms)	2300	Non applicable	600	Non applicable
Number of Slices	36	36	36	36

**Table 2:** Average (and SD) of T2* measurements at each two regions of interest for each study participant.

	Posterior Frontal (LH)		Occipital Visual (RH)	
Subject	Mean	SD	Mean	SD
1	62.35	6.4	68.63	8.3
2	61.45	4.5	53.10	2.1
3	73.65	5.3	67.10	3.2
4	75.00	6.9	74.33	7.9
5	73.85	9.6	70.07	5.3
6	76.40	7.0	76.93	7.3
7	79.70	3.1	72.65	9.9
8	70.70	6.4	65.53	6.3
9	78.00	9.3	65.00	7.7
10	73.35	6	69.47	6.3
11	71.10	7.8	64.50	7.4
12	70.90	3.0	70.40	6.3
13	68.95	7.1	67.35	7.2
14	70.50	9.2	71.50	3.8
15	72.25	9.3	71.15	9.2
16	76.50	3.8	69.45	9.7
17	70.10	8.2	75.10	4.0
18	75.25	6.3	65.20	8.8
19	66.90	4.4	63.35	2.3
20	64.00	5.4	79.75	7.4
21	70.50	4.5	75.50	5.7
Average	71.50	6.36	69.34	6.48

**Table 3:** Average extent of activation (number of active voxels) and strength of activation (peak beta values) in each TE condition.

	TE 50 ms		TE 70 ms		
	Mean	SD	Mean	SD	*p* Value
	Number of active voxels				
MI (LH)	529.3	380.4	735.9	341.8	**0.002**
PMd (LH)	111.4	117.4	142.1	121.8	**0.1**
SMA	415.1	390.7	606.3	409.9	**0.003**
SI (LH)	744.2	598.3	946.5	634.1	**0.007**
V1 (RH)	1356.5	824.8	1400.0	601.0	0.7
V2 (RH)	977.9	568.7	1102.9	530.5	0.2
	Peak beta value				
MI (LH)	1.82	1.06	3.40	1.30	**<0.001**
PMd (LH)	2.33	1.27	3.92	1.40	**<0.001**
SMA	2.24	1.06	3.53	1.36	**<0.001**
SI (LH)	2.15	1.08	3.84	1.42	**<0.001**
V1 (RH)	3.55	1.65	4.85	1.54	**<0.001**
V2 (RH)	3.18	1.87	4.61	1.66	**<0.001**

Significant *p* values at Bonferroni-adjusted *p* < 0.008 are shown in bold.

## Data Availability

Not applicable.

## Supplementary Materials

The following are available online at https://www.mdpi.com/article/10.3390/tomography7030030/s1, Figure S1: T map overlay images from one representative participant to illustrate the procedure for extracting beta values, Table S1: MNI coordinates of voxels displaying peak activation within the commonly activated cluster in the 50 ms and 70 ms TE first-level maps.
