# Supplementary material for: The Significance of Echo Time in fMRI BOLD Contrast: A Clinical Study during Motor and Visual Activation Tasks at 1.5 T
Source: Tomography. 2021 Aug 5;7(3):333–43. doi: 10.3390/tomography7030030 (PMC8396192; doi:10.3390/tomography7030030)
Supplement: Supplementary file 1 [file tomography-07-00030-s001.zip › tomography-1284048-supplementary.pdf]

Supplementary Materials

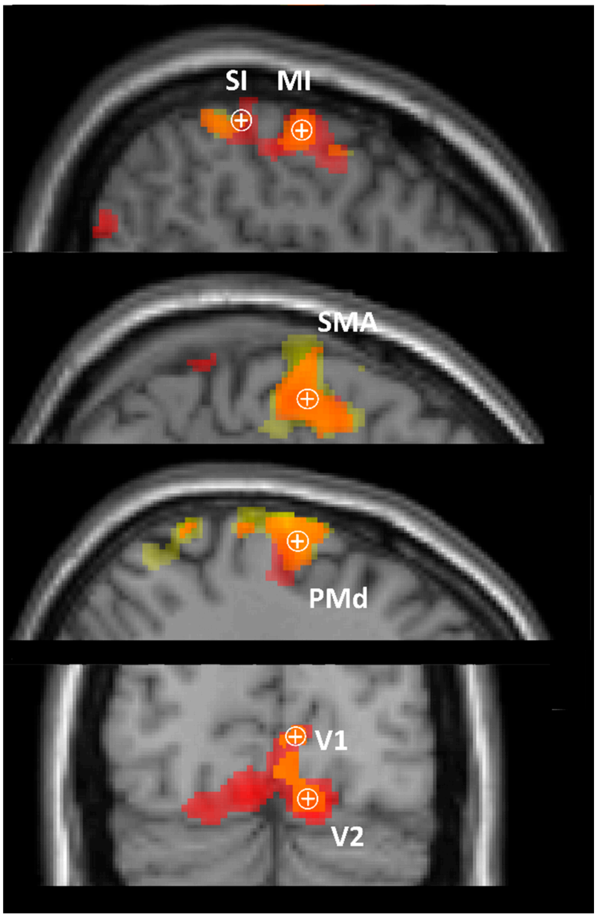

Figure S1. T map overlay images from one representative participant to illustrate the procedure for extracting beta values.

Table S1. MNI coordinates of voxels displaying peak activation within the commonly activated cluster in the 50 ms and 70 ms TE first-level maps.

S	V1 (R)	V2 (BA 18-R)	MI (L)	SI (L)	PMd (L)	SMA
**1**	–3 –86 8	−2 −91 26	−56 −9 40	−50 −22 51	−40 −6 60	−2 −5 59
**2**	–6 –100 –2	−1 −96 10	−41 −19 60	−53 −23 50	−14 −14 68	−1 −22 52
**3**	8 –88 6	8 −82 −4	−41 −16 58	−48 −22 58	−24 −16 74	0 −6 52
**4**	16 –84 4	8 −76 −8	−50 −10 52	−50 −30 54	−34 0 64	0 −4 52
**5**	10–86 6	14 −78 −14	−40 −18 56	−38 −24 56	−24 −14 72	0 −10 64
**6**	14 –82 10	4 −76 −2	−38 −18 57	−41 −23 57	−30 −22 68	--
**7**	−4 −98 −4	12 −76 −12	−40 −18 56	−44 −32 60	−34 −10 68	0 4 54
**8**	12 −88 6	12 −74 −4	−34 −22 54	−52 −28 54	−36 −10 64	0 4 52
**9**	8 −94 8	16 −76 −16	−50 −14 50	−52 −30 42	−34 −6 64	0 −8 56
**10**	−2 −84 8	14 −76 −6	−40 −20 58	−34 −36 62	−26 −11 68	2 −8 58
**11**	8 −82 8	18 −76 −14	−42 −14 60	−48 −32 58	−30 −6 68	2 6 54
**12**	−4 −82 2	8 −80 −8	−42 −12 58	−48 −18 54	−40 −20 67	0 −4 54
**13**	10 −86 4	−6 −84 −14	−40 −18 58	−54 −16 52	−40 −14 64	0 0 50
**14**	12 −78 2	12 −80 −14	−36 −18 56	−44 −24 60	−36 −12 60	0 −4 64
**15**	10 −80 12	6 −70 −4	−38 −24 56	−40 −30 52	−28 −12 62	4 4 65
**16**	6 −76 10	−10 −78 −12	−52 −4 42	−58 −22 38	−30 −14 70	2 −2 60
**17**	10 −78 8	12 −68 −8	−46 −20 64	−46 −24 64	33 −5 66[1]	0 −8 56
**18**	10 −74 4	10 −78 −12	−44 −14 58	−51 −28 54	−34 −10 68	−2 −6 68
**19**	4 −82 2	0 −84 −4	−52 −4 46	−56 −28 50	−20 −14 74	2 −8 68
**20**	2 −74 12	6 −82 −8	−46 −18 56	−40 −28 52	−36 −12 68	0 −6 56
**21**	10 −74 6	−4 −74 −8	−44 −16 60	−44 −34 62	−34 −10 68	−2 −8 54

[1]Right hemisphere. R: right hemisphere; L: Left hemisphere; S: Subject.
